# Randomised, Controlled, Assessor Blind Trial Comparing 4% Dimeticone Lotion with 0.5% Malathion Liquid for Head Louse Infestation

**DOI:** 10.1371/journal.pone.0001127

**Published:** 2007-11-07

**Authors:** Ian F. Burgess, Peter N. Lee, Geraldine Matlock

**Affiliations:** 1 Medical Entomology Centre, Insect Research & Development Limited, Royston, United Kingdom; 2 P. N. Lee Statistics and Computing Ltd., Sutton, United Kingdom; Canadian Agency for Drugs and Technologies in Health, Canada

## Abstract

**Background:**

Malathion 0.5% has been the most prescribed pediculicide in the United Kingdom for around 10 years, and is widely used in Europe and North America. Anecdotal reports suggest malathion treatments are less effective than formerly, but this has not been confirmed clinically. This study was designed to determine whether malathion is still effective and if 4% dimeticone lotion is a more effective treatment for head louse infestation.

**Methodology/Principal Findings:**

We designed this study as an assessor blinded, randomised, controlled, parallel group trial involving 58 children and 15 adults with active head louse infestation. Each participant received two applications 7 days apart of either 4% dimeticone lotion, applied for 8 hours or overnight, or 0.5% malathion liquid applied for 12 hours or overnight. All treatment and check-up visits were conducted in participants' homes. Cure of infestation was defined as no evidence of head lice after the second treatment. Some people were found free from lice but later reinfested. Worst case, intention to treat, analysis found dimeticone was significantly more effective than malathion, with 30/43 (69.8%) participants cured using dimeticone compared with 10/30 (33.3%) using malathion (p<0.01, difference 36.4%, 95% confidence interval 14.7% to 58.2%). Per protocol analysis showed cure rates of 30/39 (76.9%) and 10/29 (34.5%) respectively. Irritant reactions were observed in only two participants, both treated with malathion.

**Conclusions/Significance:**

We concluded that, although malathion liquid is still effective for some people, dimeticone lotion offers a significantly more effective alternative treatment for most people.

**Trial Registration:**

Controlled-Trials.com ISRCTN47755726

## Introduction

In a previous study [1] we demonstrated 4% dimeticone lotion was effective to eliminate head louse infestation from 70% of cases and equivalent in activity to 0.5% phenothrin liquid. Dimeticone lotion is now a licensed medicinal over the counter product in the UK. A later study [2] comparing “bug-busting” (Community Hygiene Concern, London) with a single application of insecticide found malathion 17% successful. 0.5% malathion liquid is a treatment of choice for many Primary Care Trusts and minor ailment schemes and is a first choice insecticide recommendation on the Clinical Knowledge Summaries (formerly PRODIGY) online guidance for head louse treatment. [3]

We have been aware that resistance to malathion in head lice has been spreading in the UK since it was first identified from Brighton in 1995. [4] Malathion resistance is now found in most parts of the country [5]–[7] but was not identified in a recent study conducted in Wales to investigate resistance to insecticides there. [8]

We have performed a controlled trial, analysed by intention to treat, comparing the efficacy of two applications 7 days apart of either 4% dimeticone lotion or 0.5% malathion liquid. The treatment interval of 7 days complies with the marketing authorisation approval for 4% dimeticone lotion and the recommendations set out in the British National Formulary. [9]

## Methods

The protocol for this trial and supporting CONSORT checklist are available as supporting information; see Checklist S1 and Protocol S1.

### Participants

Participants were recruited via local newspaper and radio advertising. Families responding to advertising were sent a Participant Information Booklet (PIB) by post. Those who then wished to enrol telephoned again to request a home visit, usually within 24 hours. Trained investigators used a standard protocol to examine participants for head lice using a plastic detection comb (“PDC”, KSL Consulting, Denmark). If lice were found, and the participant was eligible, a signed consent and assent procedure was followed. Other household members were offered examination and invited to join if eligible.

All enrolled participants provided baseline data on age, gender, hair characteristics, and previous pediculicide use. The lower age limit was 6 months in conformity with the licence for both products; there was no upper limit. Treatments and assessments were conducted in the home. No payment was offered for participation. Ineligible infested household members were provided with 4% dimeticone lotion.

Participants were required to confirm their availability for the duration of the study (14 days following the first treatment) in order to be included in the study. Exclusion criteria were:

- Known sensitivity to any ingredients in the treatments.
- Secondary bacterial infection of the scalp (e.g. impetigo) or any long term scalp condition other than head louse infestation (e.g. psoriasis of the scalp).
- Use of other head louse products within the previous two weeks.
- Use of hair bleach, colour, or permanent wave products, within the previous four weeks.
- Treatment with the antibiotics Co-Trimoxazole or Trimethoprim within the previous four weeks, or taking such a course at the time of enrolment.
- Pregnant or nursing mothers.
- Participation in another clinical study within 1 month before entry to this study.
- Previous participation in this clinical study. [10]

### Ethics

Prospective participants who wished to participate reported that they understood the purpose and requirements of the study outlined in the PIB and provided written consent. Parents or guardians provided written consent for children below 16 years, who also gave written or verbal assent witnessed by the parent/guardian. Ethical approval was granted by Hertfordshire 1 Research Ethics Committee (EudraCT 2006-004136-73).

The study was conducted in conformity with the principles of the Declaration of Helsinki and of European Union Directive 2001/20/EC.

### Interventions

Dimeticone 4% lotion was supplied in 150 ml bottles (Hedrin® 4% lotion, Thornton & Ross Ltd, Huddersfield, UK) and 0.5% malathion liquid in 200 ml bottles (Derbac-M liquid, SSL International, Manchester, UK). Both products were applied to dry hair, using enough to thoroughly moisten the hair and scalp. Investigators applied the products evenly through the hair using their fingers. Treatments were applied to the full hair length and left to dry naturally. [1] The same regimen was repeated 7 days later.

Participants were provided with non-medicated, conditioner free shampoo to ensure all treatments were washed off using the same preparation. Carers were advised of the earliest time treatment should be removed, usually the following morning, and asked not to use louse combs, other form of head louse treatment during participation, and not to divulge the treatment to assessors to maintain blinding. Most participants had previously used one or both preparations so it was impossible to blind them to treatment. However, when asked about the most recent previous treatment it was found only five had used a malathion product, between 2 months and 3 years previously, four of whom were allocated dimeticone and one malathion. Compliance with the protocol was assessed by retrospective questionnaire at each assessment.

### Objectives

The study was designed to compare the efficacy of 4% dimeticone lotion with 0.5% malathion liquid with sufficient power to be able to determine if activity against head lice of either product was superior to the other.

### Outcomes

The primary outcome measure was elimination of head lice using two applications of treatment. All participants were examined by dry detection combing, using the “PDC” comb, on days 2, 6, 9, and 14 after the first application of treatment unless they were lost to follow up. Examinations were performed using the comb systematically across the whole scalp. Examinations on days 2, 6, and 9 were limited to 2–3 strokes of the comb on each section, intended to provide diagnostic snapshot data of the status of infestation, because more prolonged combing could have become an additional intervention. A more extensive examination was made on day 14 to try to ensure no lice were present. “Cure” was defined as no lice after the second application of treatment, on days 9 and 14.

Previous experience showed a high risk of reinfestation after cure. [1] Knowledge of family circumstances helped identify some reinfestation risks but for statistical purposes we arbitrarily specified criteria for reinfestation as a) no adult lice or third stage nymphs found after the first treatment; and b) on days 9 or 14, no more than two adult lice or third stage nymphs and no younger nymphs found during combing. We acknowledge these criteria could give false outcomes either way but from use over several studies we believe they address the issue of reinfestation without presenting an unreasonably optimistic view of the product efficacy.

Any participant not fitting the cure or reinfestation after cure criteria was categorised as a treatment failure.

### Sample size

A sample size of 31 per group was estimated to have at least 80% power to detect (with 95% confidence) a difference of 35% between the success rates for 4% dimeticone lotion and 0.5% malathion liquid, based on a 70% success rate for dimeticone 4% lotion and evidence suggesting lower success rates with 0.5% malathion liquid, of about 19%–35%. [1], [2], [11] The planned sample sizes of 34 per group made some allowance for drop out.

### Randomization—Allocation concealment

Treatments were randomised using a computer generated list in balanced blocks of 10. Allocation was by inclusion of instruction sheets in numbered sealed envelopes issued in batches of ten to each investigator. A duplicate set was made in the event individual code breaking was required. At enrolment treatment was allocated using the next available number held by the investigator. As randomisation was by individual, household members could receive different treatments. In the event, 73 participants were treated. After the completion of the study, an administrative error had occurred whereby the wrong treatment instructions were included in some of the envelopes. This meant that seven participants originally scheduled to have 0.5% malathion in the randomisation scheme were actually allocated dimeticone 4%. This was discovered during analysis when it was found the individually numbered bottles allocated to some participants did not match the treatment group expected from the randomisation schedule. We knew what treatment a participant had received because their study number and initials were written on the bottle label by the investigator giving treatment. The result was 43 participants were given 4% dimeticone and 30 participants were given 0.5% malathion. Because this error did not compromise blinding of either treatment allocation by investigators in the field, or the assessors assigned to perform the checkups, the viability of the study was not considered to have been impaired, particularly as such a distribution could have occurred naturally as a result of some investigators using only part of their allocation of numbered envelopes. The power to detect a 35% difference with these group sizes was actually very similar to that for the original design.

### Blinding

This study was single blinded because the physical forms of the products are sufficiently different for double blinding to be impractical. Most participants had used one or both preparations previously so it was impossible to blind them to the treatment being used. Different investigators from those applying treatment, blinded to the allocation, performed assessments using “PDC” louse detection combs. Lice found during assessments were removed and fixed to the case record using clear tape. These were later examined under a microscope by another investigator, also blinded to treatment, to determine their developmental stage and if mature, their gender.

### Statistical methods

For presence/absence variables, Fisher exact tests were used. Differences in success rates between the treatments were quantified by the 95% confidence interval, calculated using a normal approximation to the binomial distribution.

For graded or semi-continuous variables, Kruskal-Wallis analysis of variance was used. As there were only two groups, this was equivalent to using the Mann-Whitney U test.

## Results

### Participant flow

**Figure 1:**
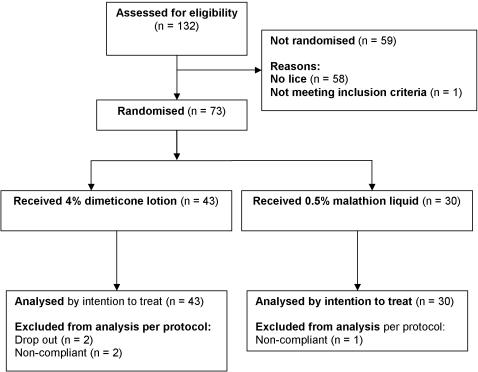
CONSORT flowchart of participants through the study

73 people from 32 families received dimeticone lotion (43) or malathion liquid (30) and 68 (93.2%) participants (39 dimeticone, 29 malathion) completed the trial (Figure 1). There were 4 withdrawals from the dimeticone group: 1 dropped out after one follow up for family reasons, as did the single drop out from the malathion group. Three others from the dimeticone group failed to complete the study, not keeping any appointments after the first assessment on day 2. These were treated as cases lost to follow up. All other participants had complete data sets, with two treatments given 7 days apart and post treatment assessments conducted on days 2, 6, 9, and 14 after the first treatment.

### Recruitment

The study was conducted between October and December 2006 when consent to participate was obtained for 58 children aged 1 to 13 and 15 adults.

### Baseline data

There was no significant difference between the groups in respect of age, gender, intensity of infestation, hair length, thickness (density), degree of curl, or whether dry/greasy (Table 1). Hair characteristics may influence the quantity of treatment required or ease of application, e.g. dry hair and closely spaced (thick) hair both require more product than greasy or thin (widely spaced or sparse) hair and can increase the difficulty of spreading some products evenly over the hair.

**Table 1:** Baseline demographic characteristics of participants (ITT)

Data subgroup	4% dimethicone	0.5% malathion
	(n = 43)	(n = 30)
Age in years:		
range	1 to 48	3 to 48
mean	13.8	12.8
median	8	9.5
Gender:	number of participants (%)	number of participants (%)
male	9 (20.9)	3 (10.0)
female	34 (79.1)	27 (90.0)
Infestation:		
light	14 (32.6)	11 (36.7)
medium	18 (41.9)	10 (33.3)
heavy	11 (25.6)	9 (30.0)
Hair length:		
close cropped	4 (9.3)	1 (3.3)
above ears	4 (9.3)	2 (6.7)
ears to shoulders	11 (25.6)	6 (20.0)
below shoulders	24 (55.8)	21 (70.0)
Hair thickness:		
fine	4 (9.3)	7 (23.3)
average	28 (65.1)	16 (53.3)
thick	11 (25.6)	7 (23.3)
Hair curl:		
straight	26 (60.5)	17 (56.7)
wavy	14 (32.6)	9 (30.0)
curly	3 (7.0)	4 (13.3)
Hair oiliness:		
dry	3 (7.0)	2 (6.7)
normal	40 (93.0)	28 (93.3)

### Outcomes and estimation

Post treatment examinations found 25 cures and 5 cases of reinfestation after cure with dimeticone and 9 and 1 respectively for malathion. If the 5 drop outs were taken as treatment failures (worst case analysis) the data provided positive outcomes of 30/43 (69.8%) for dimeticone and 10/30 (33.3%) for malathion with a significant difference of 36.4% (14.7% to 58.2%, p<0.01). Per protocol population positive outcomes were 30/39 (76.9%) for dimeticone and 10/29 (34.5%) for malathion with a more highly significant (p<0.001) difference of 42.4% (20.7% to 64.2%).

The advantage of dimeticone over malathion was found in all the data subgroups analysed (apart from those experiencing adverse events), those showing statistically significant differences being shown in Table 2. The most marked difference between the treatments was for participants with thick hair, where the rate of cure was only 1/7 (14.3%) for 0.5% malathion liquid as against 11/11 (100.0%) for 4% dimeticone lotion (p<0.001), which may be a reflection of the greater difficulty of spreading the malathion treatment evenly through thick hair.

**Table 2:** Comparison of rates of positive outcome by treatment and data subgroup

Data subgroup	4% dimeticone		0.5% malathion		
	n/N	%	n/N	%	p
All participants	30/43	69.8	10/30	33.3	<0.01
Sex -					
males	7/9	77.8	1/3	33.3	NS
females	23/34	67.7	9/27	33.3	<0.05
Infestation -					
light	12/14	85.7	7/11	63.6	NS
moderate	11/18	61.1	2/10	20.0	<0.1
heavy	7/11	63.6	1/9	11.1	<0.05
Hair thickness -					
fine	2/4	50.0	3/7	42.9	NS
medium	17/28	60.7	6/16	37.5	NS
thick	11/11	100.0	1/7	14.3	<0.001
Hair curl -					
straight	17/26	65.4	5/17	29.4	<0.05
wavy or curly	13/17	76.5	5/13	38.5	<0.1
Hair type -					
dry	3/3	100.0	1/2	50.0	NS
normal	27/40	67.5	9/28	32.1	<0.01
Adverse events seen -					
no	27/38	71.1	4/21	19.1	<0.001
yes	3/5	60.0	6/9	66.7	NS
Other family member in study -					
no	5/5	100.0	2/5	40.0	NS
yes	25/38	65.8	8/25	32.0	<0.05

### Ancillary analyses

Analysis for an alternative worst case endpoint, in which re-infestation was counted as a failure, would have given success rates of 25/43 (58.1%) for 4% dimeticone and 9/30 (30.0%) for 0.5% malathion, with the difference in rates estimated as 28.1% (6.1% to 50.2%). This difference was less significant (p<0.05) than when cases of reinfestation were included.

We considered the possibility that the incorrectly randomised participants may have shown a difference of response from the rest of the participants. We found the intention to treat cure rate 4/7 (57%) and the per protocol rate 4/6 (67%) do not differ markedly from the overall response to 4% dimeticone. Details of the outcomes for this sub-group are available as supporting information; see Table S1.

We also checked the effect of different treatments given to members of a family group. Of 32 households taking part, 22 had more than one participant, and 10 families received either only dimeticone or only malathion. Four others had members on different treatments but all people in these households were either cured or treatment failures. Variation in response to the two treatments could be found in only 8 households and the outcomes of those treatments are shown in Table 3. Only these households could effectively contribute to a test of treatment difference, which limited the power to detect a difference. Nevertheless a significantly (p<0.01) higher success rate was found for 4% dimeticone.

**Table 3:** Treatment outcome in households with different outcomes for individuals using different treatments

Family	Number of participants			Treatment outcome			
				Dimeticone		Malathion	
	Total	Dimeticone	Malathion	Cure	Failure	Cure	Failure
106	3	1	2	1	0	0	2
107	3	1	1	2	0	0	1
115	6	4	2	4	0	0	2
116	3	1	2	1	0	0	2
117	4	3	1	2 *	1	0	1
120	2	1	1	1	0	0	1
122	4	3	1	1 *	2	1	0
134	3	1	2	1	0	1	1

* Cases of reinfestation after cure

Dimeticone has not been considered to prevent eggs from hatching whereas malathion has always been thought ovicidal. We examined lice collected on days 2 and 6 for first and second stage nymphs. We found a non-significant (p = 0.28) difference for inhibition of egg hatching, with no nymphs hatched after the first treatment on 21/43 (49%) participants with dimeticone and 10/30 (33%) with malathion, a difference of 16% (−7% to 38%).

Laboratory tests show dimeticone to be completely pediculicidal, with no recovery, provided lice have adequate contact with the product. Consequently, a reasonable expectation would be that all lice are killed by an adequate treatment, with any nymphs emerging from surviving eggs killed by the second application. However, we found 9 cases categorised as failures in the dimeticone lotion group, for which we compared the outcomes and relationship to other participants. These data indicate two cases due to reinfestation from within the family, and two cases where lice were still present from day 2. The rest were all due to failure to kill eggs, with delayed hatching in some cases. Details of the outcomes for this sub-group are available as supporting information; see Table S2. As we found only a few nymphs we could not say whether these eggs were delayed in their development by the action of the treatment, i.e. they took longer than normal to hatch, or whether this was because some eggs inherently take longer to hatch.

Questionnaires showed investigators rated 4% dimeticone easier to apply (p<0.01), and easier (p<0.01) and quicker (p<0.1) to work into the hair. The participants rated the products feeling similar after application, but dimeticone as less odorous (p<0.001), easier to wash out (p<0.05) and leaving the hair feeling softer when dried (p<0.05). Both products were non-irritant to the carers' hands and generally left the hair easy to comb. Those given dimeticone were significantly more inclined to use the product again than those using malathion (97% vs 31%, p<0.001)

### Adverse events

The safety evaluation found 5 adverse events in 43 participants using dimeticone and 9 in 30 people using malathion. There were no serious adverse events. Details of the adverse events are available as supporting information; see Table S3. No difference was seen between groups in number of adverse events, severity of adverse events, relationship to study treatments, or action taken regarding them (no participant had treatment interrupted for an adverse event). The two participants with treatment related events were both in the malathion group. Both experienced itching or irritation of the scalp or neck during the treatment.

## Discussion

### Interpretation

We have found that 4% dimeticone lotion is superior in efficacy to 0.5% malathion liquid using two applications a week apart, and dimeticone was at least as effective (70%) as previously.[1] Additionally, using exploratory analyses, we found a non-significant trend suggesting 4% dimeticone lotion is more ovicidal than 0.5% malathion liquid, and more active against louse eggs than previously thought. However, the small sample size may have been a source of imprecision so further investigation of the ovicidal effect is required.

### Generalizability

This is the second study investigating activity of 4% dimeticone lotion with the primary outcome showing the same efficacy as obtained previously (70%). [1] The positive outcome for 0.5% malathion achieved using two applications was nearly twice the efficacy achieved by Hill et al. [2] using a single application and close to our estimation based on published data. We believe that these are true representations of the current activity of the preparations when used correctly, both products having been used with equal thoroughness, although the malathion product was slightly more difficult to apply. Therefore, we refute the suggestion made by the London New Drugs Group [12] that in our earlier study we applied dimeticone more rigorously because it was not possible to blind application of the products, leading to bias. [1] If that were true, it is unlikely the 0.5% phenothrin liquid comparator would have demonstrated a greater efficacy (75%) than dimeticone, especially as phenothrin has been shown to have low efficacy in other studies. [13], [14]

The difference in treatment outcomes may be attributable to resistance to malathion. It is possible that these results may not be generalizable to all malathion preparations as it is believed alcoholic malathion products may have greater potency and the terpenoids included in some alcoholic lotions contribute towards the activity of the product. [13], [15], [16] However, recent *ex vivo* data, using lice collected from the same community as our study group and then exposed to insecticide *in vitro*, suggest that head lice have developed resistance to terpenoids and that alcoholic 0.5% malathion lotions are no more effective than the 0.5% malathion aqueous liquid used in our study. In that work we immersed batches of lice in either alcoholic or aqueous malathion products as previously described [15] and observed the effects over 2 hours. Lice treated using alcoholic 0.5% malathion with terpenoids showed only 23% mortality compared with 47% for those exposed to the aqueous product. In 1995, at the time resistance to malathion was first identified, similar tests showed 100% kill using alcoholic malathion and over 90% using the aqueous preparation. All lice tested *ex vivo* in the same way but using dimeticone were immobilised without recovery within 2 minutes (Brunton ER, Burgess IF, personal communication). Consequently, we conclude that 4% dimeticone lotion is likely to prove more reliably effective than malathion products in current consumer use.

We found a similar low incidence of treatment related adverse events to previously. For dimeticone this was a matter of avoiding fluid flowing near to eyes. With 0.5% malathion we did find two cases of scalp stinging where louse bites appeared irritated by the product possibly attributable to the cetyl stearyl alcohol (Lanette wax SX) component. However, the incidence was lower than with 0.5% phenothrin liquid, [1] which has the same vehicle but with addition of diethylene glycol and dimethyl phthalate, both of which have the potential to irritate. Nevertheless, even the low incidence of irritation with 0.5% malathion liquid, which contains less cetyl stearyl alcohol than most conditioners, indicates that this material applied to louse bitten skin can exacerbate the itching resulting from infestation.

### Overall evidence

Previously we experienced problems with reinfestation, which we presumed originated from other household members unable to participate and who were not adequately treated. [1] Several cases of reinfestation were classified as treatment failures. In this study we addressed this issue, by enrolment of young siblings and offering treatment to non-participants. Contrary to expectation we did not reduce the recorded rate of reinfestation. Previously 6/127 (5%) positive treatment outcomes using dimeticone were classified as reinfestation after cure compared with 5/43 (12%) in this study. Since 3 of the 5 cases were in households where reinfestation could not have occurred it appears the risk of reinfestation from outside the family, and the control of the study, may be greater than between siblings.

Similarly we identified apparent cases of more extensive reinfestation amongst the treatment failure group. In one case this also appeared to come from outside the family. However, the majority of treatment failures were due to one or two nymphs that had apparently hatched after day 8, raising questions about how long head louse eggs take to hatch. This issue has been discussed by numerous authors [see 17] but we know of no published experimental data relating specifically to head lice (not clothing/body lice) maintained at scalp temperature (approximately 34–36° Celsius) and the only data we have seen are in a PhD thesis that shows skin temperature enables head louse eggs to hatch in 6–7 days.[18] Consequently, instructions for pediculicides vary from country to country, often based on *in vitro* clothing/body louse data that may not be an appropriate comparison. Furthermore instructions are often vague, suggesting a second treatment after 7–10 or 7–12 days, which places a considerable burden of judgement on the consumer. Therefore, we think it a matter of importance that new data on the rate of head louse egg development at scalp temperature should be obtained.

Since its launch to public sales in January 2006, 4% dimeticone lotion has become the market leading licensed head louse treatment in the UK with a share (by value) of 43% (Information Resources, Inc., 4 weeks to 27^th^ Jan 2007), fulfilling the initial estimation that it would appeal to consumers who wished to use a head louse treatment product free from neurotoxic insecticides and with no odour. A similar response to the preparation has occurred in each of the other European countries where the product is available as a medical device. This has occurred as a result of public attitude to the preparation rather than specifically due to clinical evidence recommendations from health care practitioners as no meta-analysis evaluation has yet been conducted on the product. Given that most of the evidence for other active materials is now not only relatively old but also possibly outdated by the impact of resistance, in the absence of extensive up to date data it may be difficult to conduct an analysis of evidence for pediculicides in general that has true clinical meaning and applicability.

## Supporting Information

Checklist S1 CONSORT Checklist
(0.05 MB DOC)

Protocol S1 Trial Protocol
(0.28 MB DOC)

Table S1 Treatment outcome for incorrectly randomised participants
(0.03 MB DOC)

Table S2 Analysis of cases of treatment failure following 4% dimeticone lotion treatment
(0.04 MB DOC)

Table S3 Adverse events as recorded on the case report form
(0.05 MB DOC)
