# Supplementary material for: Randomised, Controlled, Assessor Blind Trial Comparing 4% Dimeticone Lotion with 0.5% Malathion Liquid for Head Louse Infestation
Source: PLoS One. 2007 Nov 7;2(11):e1127. doi: 10.1371/journal.pone.0001127 (PMC2043492; doi:10.1371/journal.pone.0001127)
Supplement: Protocol S1 — Trial Protocol (0.28 MB DOC) [file pone.0001127.s002.doc]

A randomised, controlled, assessor-blind,

clinical trial to demonstrate superiority of Hedrin 4% dimeticone lotion compared with Derbac-M 0.5% malathion aqueous liquid in the treatment of head lice

#### CTMK05

Version 1.0, 10 July 2006

A randomised, controlled, assessor-blind,

clinical trial to demonstrate superiority of Hedrin 4% dimeticone lotion compared with Derbac-M 0.5% malathion aqueous liquid in the treatment of head lice

**Version Number:** Version 1.0, 10 July 2006

**Product Name:** Hedrin 4% lotion

**Country:** UK

**Clinical Study Number:** CTMK05

**Chief/Principal Investigator:** Ian Burgess – Director

Medical Entomology Centre, Insect Research and Development Ltd, Cambridge House, Barrington Road, Shepreth, Royston, Hertfordshire, SG8 6QZ

Tel: 01763 263011

Fax: 01763 263022

**Clinical Research Manager:** Elizabeth Kidman – Clinical Research Manager Address and contact numbers as above.

**Research nurse/Co-investigator** Geraldine Matlock – Research Nurse

Address and contact numbers as above

**Research Physician/Co-investigator:** Dr Paul Silverston

Oakfield Surgery, Vicarage Road, Newmarket, Suffolk, CB8 8HP

Tel: 01638 662018

Fax: 01638 660294

**Statistician:** Peter Lee – Director

P. N. Lee Statistics and Computing Ltd, Hamilton House, 17 Cedar Road, Sutton, Surrey, SM2 5DA

Tel: 0208 642 8265

Fax: 0208 642 2135

**Clinical Research Manager/** Stephen Skilleter

**Study Sponsor Representative:** Thornton & Ross Ltd, Linthwaite, Huddersfield,

HD7 5QH

Tel: 01484 842217

Fax: 01484 847301

**Estimated Start Date:** September 2006

**Estimated Completion Date:** December 2006

"Notice: Part or all of the information contained herein may be unpublished material and should be treated as the confidential property of Thornton & Ross Ltd, not to be divulged to unauthorised persons in any form, including publications and presentations, without the expressed written consent of Thornton & Ross Ltd."

# Investigator's Agreement

We have read this Thornton & Ross Ltd approved protocol, number CTMK05, dated 10 July 2006, entitled "A randomised, controlled, assessor-blind, clinical trial to demonstrate superiority of Hedrin 4% dimeticone lotion compared with Derbac-M 0.5% malathion aqueous liquid in the treatment of head lice”, and have discussed it to our satisfaction with Thornton & Ross Ltd.

We agree to conduct the study according to this protocol and to comply with its obligations, subject to ethical and safety considerations.

We understand that should we be in breach of any of the terms of this protocol, or if we are negligent, that Thornton & Ross Ltd, would not be held responsible for any resulting losses, damages, costs and expenses of whatever kind made by or on behalf of a participant.

Chief Investigator:

________________________Dated ___/___/___

Ian Burgess

Clinical Trial Manager:

________________________Dated ___/___/___

Elizabeth Kidman

Statistician: ________________________Dated ___/___/___

Peter Lee

Research Physician/Co-Investigator: ________________________Dated ___/___/___

Dr Paul Silverston

Thornton & Ross Ltd

Clinical Research Manager: ________________________Dated ___/___/___

Stephen Skilleter

Should the decision be made by Thornton & Ross Ltd to terminate the study at any time, such decision will be communicated to the Investigator in writing, and appropriate arrangements will be agreed upon and specified in writing. Conversely, should the Investigator decide to withdraw from execution of the study he/she will communicate immediately such decision in writing to Thornton & Ross Ltd.

**Contents**

[Investigator's Agreement 3](#__RefHeading___Toc140296663)

[1. Introduction 6](#__RefHeading___Toc140296664)

[1.1 Summary of the Study 6](#__RefHeading___Toc140296665)

[1.2 Rationale 7](#__RefHeading___Toc140296666)

[1.3 Aims (Objectives) 8](#__RefHeading___Toc140296667)

[1.4 Design in Brief 8](#__RefHeading___Toc140296668)

[2. Materials and Methods 9](#__RefHeading___Toc140296669)

[2.1 Participant Selection 9](#__RefHeading___Toc140296670)

[2.1.1 Total Numbers of Participants and Study Duration 9](#__RefHeading___Toc140296671)

[2.1.2 Inclusion Criteria 9](#__RefHeading___Toc140296672)

[2.1.3 Exclusion Criteria 9](#__RefHeading___Toc140296673)

[2.2 Clinical Supplies and Materials 10](#__RefHeading___Toc140296674)

[2.2.1 Physical Forms of the Study Supplies 10](#__RefHeading___Toc140296675)

[2.2.2 Packaging and Labelling 10](#__RefHeading___Toc140296676)

[2.2.3 Care of Supplies 11](#__RefHeading___Toc140296677)

[2.2.4 Study Materials 11](#__RefHeading___Toc140296678)

[2.2.5 Compliance 11](#__RefHeading___Toc140296679)

[2.3 Procedures and Investigations 11](#__RefHeading___Toc140296680)

[2.3.1 Treatment Regimen/Allocation 11](#__RefHeading___Toc140296681)

[2.3.2 Randomisation 11](#__RefHeading___Toc140296682)

[2.3.3 Study Methodology 11](#__RefHeading___Toc140296683)

[2.3.3.1 Pre-recruitment 11](#__RefHeading___Toc140296684)

[2.3.3.2 Recruitment (Day 0) 12](#__RefHeading___Toc140296685)

[2.3.3.3 Follow up Assessments (Days 2, 6 and 9) 14](#__RefHeading___Toc140296686)

[2.3.3.4 Follow up Treatment (Day 7) 15](#__RefHeading___Toc140296687)

[2.3.3.5 Final Assessment (Day 14) 15](#__RefHeading___Toc140296688)

[2.3.4 Concomitant Medication 15](#__RefHeading___Toc140296689)

[2.3.5 Adverse Events 16](#__RefHeading___Toc140296690)

[2.3.6 Serious Adverse Events 16](#__RefHeading___Toc140296691)

[2.3.7 Withdrawals 16](#__RefHeading___Toc140296692)

[3. Analysis and Reports 17](#__RefHeading___Toc140296693)

[3.1 Definition of End Points 17](#__RefHeading___Toc140296694)

[3.1.1 Safety 17](#__RefHeading___Toc140296695)

[3.1.2 Efficacy 17](#__RefHeading___Toc140296696)

[3.2 Definition of Populations to be analysed 18](#__RefHeading___Toc140296697)

[3.3 Proposed Primary and Secondary Analyses 18](#__RefHeading___Toc140296698)

[3.4 Statistical Methods 19](#__RefHeading___Toc140296699)

[3.5 Final Study Report 19](#__RefHeading___Toc140296700)

[4 Administrative Procedures 20](#__RefHeading___Toc140296701)

[4.1 Regulatory Documentation 20](#__RefHeading___Toc140296702)

[4.2 Ethics Committee Approval 20](#__RefHeading___Toc140296703)

[4.3 Informed Consent 20](#__RefHeading___Toc140296704)

[4.4 Insurance Policy 20](#__RefHeading___Toc140296705)

[4.5 Compensation 21](#__RefHeading___Toc140296706)

[4.6 Investigator's Responsibilities 21](#__RefHeading___Toc140296707)

[4.7 Curriculum Vitae 22](#__RefHeading___Toc140296708)

[4.8 Case Record Form 22](#__RefHeading___Toc140296709)

[4.9 Monitoring of the Study 22](#__RefHeading___Toc140296710)

[4.10 Quality Assurance 22](#__RefHeading___Toc140296711)

[4.11 Protocol Appendices 23](#__RefHeading___Toc140296712)

[4.12 Protocol Amendments 23](#__RefHeading___Toc140296713)

[4.13 Publication Policy 23](#__RefHeading___Toc140296714)

[4.14 Early Termination of the Study 23](#__RefHeading___Toc140296715)

###### Appendices

Appendix 1 Declaration of Helsinki.

Appendix 2 European (CPMP) Guidelines on Good Clinical Practice for Trials on Medicinal Products.

# Introduction

## 1.1 Summary of the Study

Title: A randomised, controlled, assessor-blind,

clinical trial to demonstrate superiority of Hedrin 4% dimeticone lotion compared with Derbac-M 0.5% malathion aqueous liquid in the treatment of head lice

**Chief Investigator:** Ian F Burgess

**Estimated Study Start:** September 2006

**Estimated Study Finish:** December 2006

**Participants:** Sixty-eight (68) evaluable participants will be recruited to the study. Thirty-four (34) participants will be treated with the Hedrin 4% lotion and thirty-four (34) with Derbac-M liquid.

**Type:** Children (aged 6 months and over) and adults who, upon inspection, are found to have live head lice.

**Products:** Hedrin 4% dimeticone lotion (Hedrin 4% lotion) and Derbac-M 0.5% malathion aqueous liquid (Derbac-M liquid).

**Methods of Application:** Hedrin 4% lotion: The product will be applied directly to dry hair. Sufficient product will be applied to saturate the hair and scalp. The product will be left in place for 8 hours (or overnight) before being shampooed and rinsed off with water. The product will be reapplied at Day 7.

Derbac-M liquid: The product will be applied directly to dry hair. Sufficient product will be applied to saturate the hair and scalp. The product will be left in place for 8 hours (or overnight) before being shampooed and rinsed off with water. The product will be reapplied at Day 7.

**Study Design:** Participants will be recruited into the study, and randomised to one of the two treatments described above.

Participants will be assessed at Day 0 (recruitment) and then treated. A member of the Medical Entomology Centre (MEC) study team will apply the treatment. The same treatment will be reapplied on Day 7.

Participants will be assessed for the presence of live head lice on Days 2, 6, 9 and 14 (+/- 1 day). The assessors will be blind to the treatment group. Any lice found at assessments will be taped into the Case Record Form (CRF) for size analysis.

**Aims of the Study:** To demonstrate superiority of Hedrin 4% lotion over Derbac-M in the eradication of head lice and to compare the products for safety, ease of application and participant acceptability.

## 1.2 Rationale

Infestation with the human head louse (*pediculus capitis*) is of widespread concern in the UK (1, 2), especially since the appearance of strains of head lice resistant to one or more of the currently available insecticidal products (3, 4).

There is also growing public concern about the potential hazard, real and imagined, of the repeated use of these treatments. Families are aware of a resistance problem but have no way of knowing if the lice they are trying to eradicate are resistant to the product they are using. The various insecticidal compounds and formulations available all claim to be both insecticidal and ovicidal (5, 6) and public confidence diminishes when they fail to cure their family’s head lice problems.

“Non-insecticidal” measures, such as wet combing with a toiletry conditioner, are time consuming and do not guarantee success (7, data on file).

Hedrin 4% lotion, a product with the non-neurotoxic active ingredient dimeticone, was licensed as a medicine and commenced sale to the public from January 2006. However, despite the evidence of a clinical study showing its activity in comparison with the pyrethroid insecticide phenothrin (8) many health care professionals wish to see evidence of its activity in comparison with malathion, which has been treatment of choice for many Primary Care Trusts’ Pharmaceutical Advisers for several years.

This clinical study has been designed to evaluate the efficacy of Hedrin 4% lotion in comparison with the most widely used malathion product with the same contact time (Derbac-M liquid). In both cases, a repeat treatment will be applied one week later as recommended by The British National Formulary (BNF) for all currently available insecticidal treatments and in conformity to the marketing authorisation for Hedrin 4% lotion.

##### References

1. Donaldson RJ. The head louse in England: prevalence amongst school children. Health Education Council Report 1975.
2. Child Care in the Community. Guide to Good Clinical Practice. 1996.
3. Burgess IF, Brown CM, Peock S and Kaufman J. Head lice resistance to pyrethroid insecticides in Britain. British Medical Journal, 1995; 311: 752.
4. Downs AM, Stafford KA, Harvey I and Coles G. Evidence for double resistance to permethrin and malathion in head lice. British Journal of Dermatology, 1999; 141: 508-511.
5. Burgess I. Management guidelines for head lice and scabies. Prescriber 1996; 7: 87-99.
6. Burgess IF. The dermatoepidemiology of ectoparasitic infections. In: The Challenge of Dermato-epidemiology (HC Williams and DP Strachan eds) 1997, CRC. Press, Boca Raton, 295-311.
7. Roberts RJ, Casey D, Morgan DA and Petrovic M. Comparison of wet combing with malathion for treatment of head lice in the UK: a pragmatic randomised controlled trial. Lancet 2000; 356: 540-544.
8. Burgess IF, Brown CM, Lee PN. Treatment of head louse infestation with 4% dimeticone lotion: randomised controlled equivalence trial. British Medical Journal 2005; 330:1423–5.

## 1.3 Aims (Objectives)

1. To demonstrate superiority of efficacy of Hedrin 4% lotion over Derbac-M liquid in the eradication of head lice.
2. To compare Hedrin 4% lotion with Derbac-M liquid with regard to safety, ease of application and participant acceptability.

## Design in Brief

This trial will be a randomised, controlled, assessor blind, parallel group study of two topical treatments for head lice. Sixty-eight (68) evaluable participants, minimum age 6 months, who upon examination are confirmed to have head lice and who fit the selection criteria (see section 2.1.2 and 2.1.3) will be recruited to the study.

At the first visit (Day 0), verbal consent will be obtained to check for the presence of live head lice. This will be done using a fine-toothed plastic detection comb. After the preliminary assessment, participants can be enrolled to the study, provided they comply with the inclusion/exclusion criteria, and any further questions they may have are fully dealt with.

Participants (or their parents/guardians if they are aged less than 16 years) will be asked to give written informed consent and sign a Consent Form before participation in the study. There will be a separate Assent Form for children capable of giving written assent and a Verbal Assent Form for children not capable of signing their name. Children too young to give assent can be entered in the study on the consent of a parent/guardian. Both Assent Forms will be witnessed by the parent/guardian and signed by the Investigator. Consent will be sought for permission to inform the participant’s GP that their patient is taking part in the study. Any household members identified as having lice, but who are unable to participate in the study for any reason, will be offered a standard of care treatment (Hedrin 4% lotion) in order to reduce the risk of reinfestation of participants during the course of the study.

The sixty-eight (68) participants enrolled on to the study will be divided into two groups of thirty-four (34). The Investigator will assign each participant a study number, this being the next available number on a randomised treatment allocation sequence. The treatment allocations will be held in sealed envelopes and will only be opened after consent has been received.

Participants will be treated at Day 0 and Day 7 (+/- 1 day) with either Hedrin 4% lotion or Derbac-M liquid. A member of the MEC study team will apply the treatment on both occasions. Participants will be assessed on Days 2, 6, 9 and 14 (+/- 1 day at each assessment) by a member of the MEC study team who is unaware of the treatment that was applied. Any lice found at assessments will be taped into the participant’s CRF.

All adverse events will be monitored during the study (see sections 2.3.5 and 2.3.6) and all changes in concomitant illness and medication will be recorded (see section 2.3.4). A Completion/Withdrawal Form will be completed at the end of the study period.

# 2. Materials and Methods

## 2.1 Participant Selection

### Total Numbers of Participants and Study Duration

Sixty-eight (68) evaluable participants will be recruited to the study. The duration of the study will be 15 days (+/- 1 day), Day 0 being the first treatment day, with 14 days follow up.

### 2.1.2 Inclusion Criteria

1. Participants aged 6 months and over with no upper age limit.
2. Participants who upon examination, are confirmed to have live head lice.
3. Participants who give written informed consent, or if the participant is under 16 years of age whose parent/guardian gives written informed consent to participate in the study.
4. Participants who will be available for home visits by MEC study team members over the 14 days following first treatment.

### 2.1.3 Exclusion Criteria

1. Participants with a known sensitivity to any of the ingredients in Hedrin 4% lotion or Derbac-M liquid.
2. Participants with a secondary bacterial infection of the scalp (e.g. impetigo) or who have a long term scalp condition (e.g. psoriasis of the scalp).
3. Participants who have been treated with other head lice products within the previous two weeks.
4. Participants who have bleached hair, or hair that has been colour treated or permanently waved within the previous four weeks (wash in/wash out colours are acceptable).
5. Participants who have been treated with the antibiotics Co-Trimoxazole, Septrin or Trimethoprim within the previous four weeks, or who are currently taking such a course.
6. Pregnant or nursing mothers.
7. Participants who have participated in another clinical study within 1 month before entry to this study.
8. Participants who have already participated in this clinical study.

## 2.2 Clinical Supplies and Materials

### 2.2.1 Physical Forms of the Study Supplies

###### Hedrin 4% lotion contains:

Active: 4% Dimeticone

Excipient: 96% ST-Cyclomethicone

###### Derbac-M contains:

Active: 0.5% malathion

Excipients: Methylhydroxybenzoate; propylhydroxybenzoate; lanette wax SX; potassium citrate; citric acid; perfume HT 52; and purified water.

### 2.2.2 Packaging and Labelling

###### Packaging - Hedrin 4% lotion:

###### The product will be packed in plastic squeeze bottles with each container holding 150mL of material. The bottles will be made from high-density polyethylene (HDPE) dropper containers with screw caps.

###### Packaging - Derbac-M:

###### The product will be packed in glass bottles with each container holding 200mL of material. The bottles will be fitted with polyethylene caps and polypropylene faced wads.

Labelling – both products:

The bottles of both test products used in the study will be numbered and weighed on calibrated scales before use. A clinical trial label will be affixed identifying the individual bottle number and a blank section will be provided for completion of participant number and initials. Both products will be labelled with appropriate clinical trial labelling that will also state that they are “For Clinical Trial Use Only”.

### 2.2.3 Care of Supplies

All supplies used in the study must be maintained securely under the direct responsibility of the Chief Investigator or under that delegated by the Investigator.

All supplies shall be dispensed in accordance with the Investigator's direction and it is the Investigator's responsibility to ensure an accurate record of supplies issued and returned is maintained.

All supplies should be stored at room temperature (below 25^o^C), out of direct sunlight and protected from extremes of environmental conditions.

All supplies will be used only while participating in the study and returned to MEC at the end of the study for weighing before being returned to Thornton & Ross Ltd.

### 2.2.4 Study Materials

Thornton & Ross Ltd will supply all the clinical study materials required for the duration of the study. In addition, numbered CRFs will be supplied for each participant.

### 2.2.5 Compliance

All supplies used, partly used, or unused will be maintained for collection by the study monitor.

## 2.3 Procedures and Investigations

### 2.3.1 Treatment Regimen/Allocation

This is a randomised, controlled, assessor-blind parallel group study of Hedrin 4% lotion and Derbac-M liquid in the treatment of head lice. Each participant who satisfies the inclusion/exclusion criteria will be randomised into one of two groups. One group will be treated with Hedrin 4% lotion and the second group with Derbac-M liquid.

### 2.3.2 Randomisation

An independent statistician will generate the randomised treatment allocation code using computer software. The treatment allocations will be sealed in envelopes numbered sequentially on the outside with the participant number. The envelopes will only be opened once written informed consent has been obtained. The investigating team will keep a second batch of envelopes in case an adverse event, reaction or any other emergency circumstance necessitates that the code be broken.

### 2.3.3 Study Methodology

2.3.3.1 Pre-recruitment

Participants will be invited to join the study by advertisements in local newspapers. General Practitioners, Pharmacies, School Nurses and Health Visitors as well as the Primary Care Trusts and the Health Protection Agency will also be given the study information and posters to display in their public places. A telephone number will be provided and potential study recruits will be asked to telephone MEC for more information if they would like to take part in the study.

A member of the MEC study team will conduct a brief telephone interview to establish whether the person will be suitable for entry into the study. If it appears that the person will be suitable for entry, the person will be sent a detailed Participant Information Booklet (PIB). This will include a children’s section explaining what will happen if the person enters the study.

If the person wishes to enter the study, they will be invited to telephone MEC to arrange a recruitment visit at the person’s home. Potential participants must have been in receipt of the PIB for at least 24 hours before the recruitment visit takes place.

2.3.3.2 Recruitment (Day 0)

Screening:

Each potential participant will be asked for permission to assess their hair to confirm the presence of live head lice. The assessment will be made by dry combing the hair with a plastic fine-toothed head louse detection comb. Lice found during the assessment will not be removed. Other family members who give their verbal permission can also be assessed for the presence of living lice and may join the study provided they meet the inclusion/exclusion criteria.

Details will be recorded of how many people share the place of residence with the participant. Details will also be recorded of the number of people assessed and found to have lice, the number assessed and found not to have lice and the number that were not assessed. The number of people enrolled in the study will also be recorded.

Consent/Assent:

Participants and/or parents/guardians will be asked if they understand the requirements of the study and if they have any further questions concerning it. Provided they still wish to enter the study and meet the inclusion/exclusion criteria for entry, the participant or parent/guardian (when the participant is below the age of 16) will read and sign the Consent Form. The Investigator will countersign the Consent Form.

A separate Assent Form will be available for those under the age of 16 provided they are capable of signing their name. A Verbal Assent Form will be available for those under the age of 16 who are incapable of signing their name. The Investigator and the parent/guardian will countersign the Assent Forms.

Children too young to provide either written or verbal assent may be enrolled on the basis of parental consent.

The participant’s General Practitioner (GP) will be informed via a letter from the Chief Investigator that the participant is participating in the clinical study, provided participants give consent to this.

Case Record Form completion:

The following information will be recorded in the CRF:

1. **Declaration of Receipt of Informed Consent**: Confirmation that informed consent and assent (where relevant) was obtained, that a copy of the consent has been given to the participant and/or parent guardian and that the original will be retained.
2. **Identification**: Participant's initials, gender, age, date of birth.
3. **Hair Characteristics**: Characteristics will be recorded of the participant’s hair:
   1. Length: closely cropped, above ears, ears to shoulders, below shoulders
   2. Thickness: fine, medium, thick
   3. Degree of curl: straight, wavy, slight curl, tight curl
   4. Type: dry, normal, greasy
4. **Head Lice Details**: When the participant was last treated for head lice (an exact date if treated within the previous four weeks), the treatment that was used and the outcome (success or failure). The severity of the current louse infestation will be assessed using the following scale:
   1. Light infestation: lice only found after 5-6 combs of the hair
   2. Moderate infestation: single louse found on the first comb of the hair
   3. Heavy infestation: more than one louse found on the first comb of the hair
5. **Medication Current at Entry**: Any medication being taken along with the date the medication started the total dose and the reason for the medication.
6. **Medical History**: Medical history and any current illnesses will be recorded.
7. **Inclusion/Exclusion Criteria**: Confirmation that the participant meets the inclusion/exclusion criteria for entry into the study.

Randomisation:

The Investigator will carry a block of sequential numbered envelopes, which correspond to the participant numbers on the CRFs. Each envelope will contain a randomised treatment allocation. After consent has been received, the next sequential numbered envelope will be opened and the specified treatment allocated.

Treatment:

A member of the MEC study team will apply the relevant treatment to the participant and the participant number and participant initials will be added to the label of the bottle used. The remaining product will be retained and returned to MEC so that the weight can be recorded and the amount of product used calculated.

The products will be applied in the following ways:

Hedrin 4% lotion: The product will be applied directly to dry hair. Sufficient product will be applied to saturate the hair and scalp. More than one bottle can be used if required. The product will be left in place for 8 hours (or overnight) and then washed off with non-medicated frequent wash shampoo. The hair will then be rinsed with water.

Derbac-M: The product will be applied directly to dry hair. Sufficient product will be applied to saturate the hair and scalp. More than one bottle can be used if required. The product will be left in place for 8 hours (or overnight) and then washed off with non-medicated frequent wash shampoo. The hair will then be rinsed with water.

The MEC study team member will retain the bottle and any unused product remaining and return them to MEC for weighing. The participant will be reminded that a second treatment will be applied on Day 7 and will be asked not to disclose which treatment was applied to other MEC study team members who will attend to make assessments.

Care will be taken to ensure the treatment is applied at least 1 hour before the participant goes to bed so that the hair has sufficient time to dry after treatment. Hair can be dried in the usual way following hair washing after treatment.

Any household members identified as having lice, but who are unable to participate in the study for any reason, will be offered a standard of care treatment (Hedrin 4% lotion) in order to reduce the risk of reinfestation of participants during the course of the study.

Investigator Questionnaire:

The MEC study team member who applied the treatment will complete a questionnaire. This will include questions on how easy the product was to apply to the hair and scalp, how easy it was to work the product in, how long it took to apply the product and how satisfied the MEC team member was that the product saturated the hair and scalp.

2.3.3.3 Follow up Assessments (Days 2, 6 and 9)

Assessments will take place on Days 2, 6, 9 and 14 (+/- 1 day at each assessment) – see section 2.3.3.5 for Day 14. At assessments, the participant’s hair will be combed with a head louse detection comb, and any lice found will be taped (with clear tape) into the participant’s CRF. The MEC study team member that conducts the follow up assessments will be different from the MEC team member that applied the treatment at Day 0 and 7 and the participant will be reminded not to disclose which treatment was applied.

All adverse events and changes in concomitant medication will be recorded in the CRF.

- - - 1. Follow up Treatment (Day 7)

Treatment will be reapplied at Day 7 following the same procedure as at Day 0 (see section 2.3.3.2).

- - - 1. Final Assessment (Day 14)

The final assessment will take place 14 days (+/- 1 day) after the first treatment (the 15^th^ day of the study). The participant’s hair will be combed with a head louse detection comb and any lice found will be taped (with clear tape) into the participant’s CRF. The MEC study team member that conducts this follow up assessment will be different from the MEC team member that applied the treatment at Day 0 and 7 and the participant will be reminded not to disclose which treatment was applied.

At the Day 14 assessment, the participant and/or parent/guardian will complete a questionnaire on the treatment that was applied at Day 0. This will include questions on how the participant’s scalp felt, how the hair felt, how the product smelled, how easily the product washed out and whether the participant and/or parent/guardian would use the product again.

Participants who still have live lice at the end of the study will be offered a standard of care treatment (Hedrin 4% lotion).

All adverse events and changes in concomitant medication will be recorded in the CRF.

The Completion/Withdrawal Form will then be completed.

Any lice found during the course of the study will be examined under the microscope to establish the sex and/or stage of development. It is expected that few adult lice will be found during the monitoring period. The presence of small lice (nymphs) will be evidence that not all of the eggs were killed by the first treatment. Continued monitoring will enable the investigation to determine whether the presence of any lice is due to surviving lice or surviving eggs from which nymphs emerge. The occasional adult louse (1 or 2 only) found after the initial infestation is cleared will be indicative of re-infection (see section 3.1.2).

### 2.3.4 Concomitant Medication

The participant should not use any other form of pediculicide treatment while taking part in the study. If the use of such treatment occurs, the participant will be withdrawn from the study.

Other medication can be prescribed in the normal way although participants requiring Co-Trimoxazole, Septrin, or Trimethoprim should be withdrawn from the study.

All concomitant medicines should be listed in the CRF and any changes to such medicines, during the course of the study, recorded.

### 2.3.5 Adverse Events

Space will be provided specifically for recording observed and reported adverse events. All unwanted effects, whether considered to be caused by the study medication or not, will be reported to Thornton & Ross Ltd by completing the Adverse Event form.

### 2.3.6 Serious Adverse Events

If the adverse event is serious, it shall be reported immediately, by e-mail and telephone and by facsimile to the Research Physician/Co-Investigator and Thornton & Ross Ltd. A full written report will be forwarded to Thornton & Ross Ltd, by facsimile, within 3 working days.

Serious adverse events are defined as events that are fatal, life threatening, disabling or incapacitating, cause or prolong hospitalisation, overdose (of any kind, with or without symptoms), newly diagnosed cancer or clinically abnormal laboratory values (with or without symptoms).

The contacts for all serious adverse events are:

| Stephen Skilleter  Thornton & Ross Ltd  Linthwaite  Huddersfield  HD7 5QH  Tel: 01484 842217  Fax: 01484 847301  E: [phv@thorntonross.com](mailto:steveskilleter@thorntonross.com) | Dr Paul Silverston  Oakfield Surgery  Vicarage Road  Newmarket  Suffolk, CB8 8HP  Tel: 01638 662018  Fax: 01638 660294  E: [paul.silverston@gp-d83067.nhs.uk](mailto:paul.silverston@gp-d83067.nhs.uk) |
| --- | --- |

### 2.3.7 Withdrawals

Participants may be withdrawn from the study at any time for the following reasons:

Adverse Event:

The participant is withdrawn from the study by the Investigator because of an adverse event, whether or not the Investigator believes it to be serious or caused by the study medication, and provided that the Investigator considers it is in the participant's best interest to be withdrawn. There must be a corresponding entry on the Adverse Events and/or the Serious Adverse Events Form in this instance.

Non-compliance:

The participant is withdrawn because of failure to comply with the treatment regimen, or comply with the investigations as required, but is still accessible to the Investigator.

Drop Out:

The participant withdraws consent to continue in the study, but the Investigator would otherwise consider it appropriate for him/her to continue. The participant remains accessible to the Investigator.

Lost to Follow-up:

The participant, without explanation, fails to keep appointments as scheduled for study assessments and is not seen again despite the Investigator's effort (letter, telephone, home visit etc.) to re-establish contact.

Death:

All deaths will be treated as Serious Adverse Events and Thornton & Ross Ltd must be informed within 24 hours. All associated documentation must be completed within 3 working days. Full details will be required including a post-mortem examination if possible.

Lack of Efficacy:

The participant elects to withdraw, because the medication is not adequately effective.

# 3. Analysis and Reports

## 3.1 Definition of End Points

### 3.1.1 Safety

Participants will be observed and all untoward effects will be recorded, whether or not they are thought to be related to the study treatment.

Details of the recording of adverse events are shown in section 2.3.5 and 2.3.6.

### 3.1.2 Efficacy

The primary measure is the between treatment comparison of the number of participants with no evidence of active head louse infestation 14 days (+/- 1 day) after enrolment.

**Treatment Outcome Algorithm**

This algorithm has been developed by Medical Entomology Centre and P. N. Lee Statistics and Computing Ltd in order to impose limits within which the outcomes of treatment and any lice recovered can be interpreted. There will always be some results that will be difficult to define and, in these cases, the Investigator’s best judgement will be final. Any such additional judgements will be based on the knowledge of the family and household circumstances.

Cure:

No lice present on Day 9 and Day 14.

Ovicidal Failure:

Specific lice must be present at Day 9 and Day 14 as below:

- - - At Day 9 there must be no adult lice and no stage 2 or stage 3 nymphs.
    - At Day 14 there must be no adult lice or stage 3 nymphs.

This is equivalent to requiring stage 1 nymphs at Day 9 and/or stage 1 and/or stage 2 nymphs at Day 14.

Re-infestation

The following must all apply:

- - - Lice must be present at Day 9 and/or Day 14.
    - Adult lice and/or stage 3 nymphs must not be present at Day 2 or Day 6.
    - Stage 1 or 2 nymphs must not be present at Day 9 or Day 14.
    - If stage 3 nymphs are present at Day 9 there must be no stage 1 or 2 nymphs present at Day 6.
    - Stage 3 nymphs may be present at Day 9 or Day 14 although they must not exceed 2* in number.
    - Adult lice should not exceed 2* at either Day 9 or Day 14.

In addition, the following may apply:

- If adult lice are present at Day 9 Stage 1 and stage 2 nymphs may be present at Day 6.

* The number 2 is an interpretation of the limits of “occasional”.

Treatment Failure:

All other outcomes will be classed as treatment failure.

## 3.2 Definition of Populations to be analysed

The Efficacy Population:

Includes all randomised participants who are treated according to the study protocol.

"Intention-to-treat" Population:

Includes all randomised participants who are treated at least once. Premature terminations, due to treatment failure, adverse events etc., are included.

## 3.3 Proposed Primary and Secondary Analyses

1. To compare the efficacy of Hedrin 4% lotion against Derbac-M liquid in the eradication of head lice.
2. To compare Hedrin 4% lotion against Derbac-M liquid for safety, ease of use, and participant acceptability.

## 3.4 Statistical Methods

Sample Size Determination:

The study has been designed to establish the superiority of Hedrin 4% lotion over the Derbac-M liquid in clinical use. A pivotal Phase III study using Hedrin 4% lotion showed efficacy of 70% and a smaller Phase II study over 90%. No useful *in vivo* information exists on the efficacy of Derbac-M liquid. Data on 0.5% malathion, active ingredient of Derbac-M liquid, documented success rates of 95% or over around the time of regulatory approval (1971), but evidence from recent small studies conducted in the UK by other investigators leads to the expectation that the cure rate may be 50% or lower due to clinical resistance to treatment. Anecdote and local evidence of resistance to malathion suggest that lower success rates with Derbac-M liquid, of about 19%-35%, might be expected in the study.

A sample size of 31 per group will have at least 80% power to detect (with 95% confidence) a difference of 35% between the success rates for Hedrin 4% lotion and Derbac-M liquid. This will be true whether Hedrin 4% lotion shows the expected advantage (e.g. power of 80.3% for assumed success rates of 70% for Hedrin 4% lotion and 35% for Derbac-M liquid) or whether, unexpectedly, Hedrin 4% lotion is substantially inferior (e.g. power of 85.5% for assumed success rates of 50% for Hedrin 4% lotion and 85% for Derbac-M liquid). The actual sample sizes of 34 per group make allowance for dropout.

Analytical Methods:

An independent statistician (P. N. Lee Statistics and Computing Ltd) will undertake the statistical analysis.

The primary endpoint is the frequency of cure, with the difference between the two groups tested using the "intention-to-treat" population. Secondary endpoints will be the frequency of ovicidal failure and of reinfestation and the numbers of lice seen at the different assessment times. Other endpoints will be the safety of the product, the ease of use and participant acceptance.

Analyses will be conducted based on both the "intention-to-treat" and the "efficacy" populations. Difference between groups in baseline characteristics, safety, ease of use, acceptability and efficacy will be tested using Fisher's exact test for yes/no variables and the Mann-Whitney U test for ranked variables. Where analysis shows important differences in baseline characteristics between the groups, chi squared and rank tests stratified for these characteristics may also be conducted. 95% confidence limits will be presented for the difference between groups in the primary endpoint.

## 3.5 Final Study Report

A clinical report, integrating the study design and the statistical analyses will be prepared for the study and agreed by the Chief Investigator, the Statistician and the Study Managers. The Chief Investigator, the Clinical Research Manager, the Statistician, the Research Physician/Co-investigator and representatives of Thornton & Ross Ltd will sign a copy of the final study report.

# 4 Administrative Procedures

## 4.1 Regulatory Documentation

Any required legislative procedures will be undertaken before the commencement of the study. The study will not proceed without granted written approval.

This study will be conducted according to the recommendations of the European (CPMP) Guidelines on Good Clinical Practice for Trials on Medicinal Products and with the European Standard, and the EU Directive on Good Clinical Practice (2001/20/EU).

## 4.2 Ethics Committee Approval

The Chief Investigator will be required to obtain the written approval of the relevant Research Ethics Committee before commencing the study. In accordance with Good Clinical Research Practice, a copy of this approval together with the constitution of the ethics committee will be forwarded to Thornton & Ross Ltd before the release of trial supplies from Thornton & Ross Ltd.

## 4.3 Informed Consent

This study will be conducted in accordance with the principles laid down in the Declaration of the World Medical Assembly of Helsinki, as amended in Tokyo, Venice, Hong Kong, South Africa and Edinburgh (see Appendix 1).

Each participant or parent/guardian (where the participant is not legally competent) will be requested to provide written informed consent after receiving written information and a verbal explanation of what the study involves. A copy of the Consent Form will be returned to the participant and/or parent/guardian.

The Investigator will retain the original informed Consent Form, but will also complete a Declaration of Receipt of Consent Form to confirm that written informed consent was obtained. Thornton & Ross Ltd will hold the original declaration form.

As Thornton & Ross Ltd does not maintain a record of participant names, it is essential that the Investigator shall arrange for the retention of participant identification codes for at least 25 years after the completion or discontinuation of the study.

## 4.4 Insurance Policy

Thornton & Ross Ltd confirms that this specific clinical study is protected by insurance cover which provides an indemnity to the Investigators and their co-workers, subject to the Policy terms, conditions and limitations and provided always that the study is conducted and the data as reported agree to the standards fixed by the protocol. Medical Entomology Centre (through its parent Insect Research & Development Limited) maintains professional liability insurance to provide indemnity in the event of negligent acts by employees.

## 4.5 Compensation

Thornton & Ross Ltd maintains in force a "no fault" compensation insurance indemnity in accordance with the current version of the ABPI Guidelines on Clinical Trials: "Compensation for Medicine Induced Injury". In the event that the compensation on a "no fault basis" is unacceptable to the claimant, the Policy will, subject to its terms, conditions and limitations, respond to an action for legal liability arising out of this clinical study.

## 4.6 Investigator's Responsibilities

##### Good Clinical Practice

It is the responsibility of the Investigators to ensure that this study is carried out in accordance with this protocol in respect of ethical, legal and technical aspects and conforming to the European (CPMP) Guidelines on Good Clinical Practice for Trials on Medicinal Products. In this context, the Investigator shall arrange for the retention of participant identification codes for at least 25 years after completion or discontinuation of the study. Thornton & Ross Ltd will render all support necessary to assist the Investigator in discharging this responsibility.

##### Replacement of Principal Investigator

In the event of a Principal Investigator being unable to continue the study, another responsible person may be designated Investigator and documentation testifying to this will be submitted to the study monitor within 10 days. The new Investigator must be appropriately qualified and be approved by Thornton & Ross Ltd and the Local Research Ethics Committee before the study can be continued.

##### Study Report

The Chief Investigator will submit a summary study report within approximately 2 months of completion of the study. This report will include:

1. Details of the investigative procedures involved.
2. The numbers of participants entered, completed, and withdrawn from the study.
3. Deviations from the study protocol on a general basis and for individual participants, with explanations.
4. Explanations for each participant withdrawn from the study.
5. Methodology and normal ranges for laboratory investigations (where appropriate)
6. Summary of the safety and tolerance data, including details of all Adverse Drug Events (ADE) including any follow-up. Case histories of all serious ADEs or ADEs leading to withdrawal should be provided.
7. If appropriate, details of any statistical analysis carried out by the Investigators, and a summary of efficacy data including clinical observations.
8. Conclusions

A copy of the report will be forwarded to the Research Ethics Committee.

## 4.7 Curriculum Vitae

In accordance with international standards, and Good Clinical Research Practice, a signed copy of the curriculum vitae of the Principal Investigators, Research Physician/Co-Investigator, Statistician and members of the MEC study team will be provided to Thornton & Ross Ltd.

## 4.8 Case Record Form

The Investigator is required to prepare and maintain adequate and accurate case records that have been designed by Thornton & Ross Ltd to record all observations and other data pertinent to the clinical study. All CRFs should be completed in their entirety in a neat, legible manner to ensure accurate interpretation of data. Black ballpoint pen should be used to ensure the clarity of reproduced copies. Any alterations or errors to the CRF should be crossed through once only, and signed and dated by the person making the change, using black ballpoint pen.

The study monitor will examine the original CRFs at each monitoring visit and will approve them when the CRF is complete and any necessary amendments have been made. The Investigator will not sign the CRFs until the study monitor has approved them. The Investigator will retain the CRFs until completion of data collection when they will be given to the study monitor for transfer to Thornton & Ross Ltd. The Investigator will retain a copy together with other source data for his/her own files.

The CPMP Guidelines on Good Clinical Practice for Trials on Medicinal Products in the European Community require that the Investigator shall arrange for the retention of the participant identification codes for at least 25 years after the completion or discontinuation of the study. Participant files and other source data shall also be kept for the maximum period permitted by the institution but not less than 25 years.

## 4.9 Monitoring of the Study

At regular intervals during the study, a representative of the monitoring team of Thornton & Ross Ltd will visit the study centre. At each monitoring visit, the Investigator and the monitor will review study progress, compliance with the study protocol, CRF’s, and any emergent problems.

## 4.10 Quality Assurance

In accordance with Good Clinical Practice Guidelines and recommendations, Thornton & Ross Ltd may undertake an independent quality assurance audit of the clinical study and related documentation during the course of this study. The purpose of the audit is to determine whether the evaluated trial related activities were conducted, and the data were recorded, analysed and accurately reported according to the protocol, Thornton & Ross Ltd’s Standard Operating Procedures, Good Clinical Practice and the applicable regulatory requirements. At any stage during the study, the Investigator has the responsibility to make all data available to Thornton & Ross Ltd and/or relevant authority (where required) for auditing purposes. Such audits will at all times be conducted in accordance with national, legal and ethical requirements.

## 4.11 Protocol Appendices

It is specified that the appendices attached to this protocol, and referred to in the main text of this protocol, form an integral part of the protocol.

## 4.12 Protocol Amendments

Neither Thornton & Ross Ltd nor the Investigators may make any changes or amendments to this protocol, after the protocol has been agreed and signed by both parties, unless such change(s) or amendment(s) have been fully discussed and agreed by both the Investigator and Thornton & Ross Ltd. Any change or amendment agreed will be recorded in writing, the written amendment will be signed by the Investigator and by Thornton & Ross Ltd and the signed amendment will be appended to this protocol.

Any substantive changes will be forwarded to the Research Ethics Committee and to the appropriate regulatory authority for approval before implementation of the amendments.

## 4.13 Publication Policy

Submission of results for publication will not take place without prior discussion with Thornton & Ross Ltd, allowing the company sufficient time to analyse such results and provide written agreement to publication, which will not be unreasonably withheld. Thornton & Ross Ltd reserves the right to use the results and reports of this study for any purpose.

## 4.14 Early Termination of the Study

By agreement between Thornton & Ross Ltd and the Principal Investigators, the study may be terminated at any time if the recruitment rate is such that the required number of participants will not be recruited within the specified time, if the products being used are deemed to be failing unacceptably, or if any safety concerns arise.

5. Appendix 1: Declaration of Helsinki

WORLD MEDICAL ASSOCIATION DECLARATION OF HELSINKI

Ethical Principles for Medical Research Involving Human Subjects

Adopted by the 18th WMA General Assembly, Helsinki, Finland, June 1964 and amended by the:

29th WMA General Assembly, Tokyo, Japan, October 1975

35th WMA General Assembly, Venice, Italy, October 1983

41st WMA General Assembly, Hong Kong, September 1989

48th WMA General Assembly, Somerset West, Republic of South Africa, October 1996

52nd WMA General Assembly, Edinburgh, Scotland, October 2000

A. Introduction

1. The World Medical Association has developed the Declaration of Helsinki as a statement of ethical principles to provide guidance to physicians and other participants in medical research involving human subjects. Medical research involving human subjects includes research on identifiable human material or identifiable data.
2. It is the duty of the physician to promote and safeguard the health of the people. The physician’s knowledge and conscience are dedicated to the fulfilment of this duty.
3. The Declaration of Geneva of the World Medical Association binds the physician with the words, “The health of my participant will be my first consideration.” and the International Code of Medical Ethics declares that, “A physician shall act only in the participant’s interest when providing medical care which might have the effect of weakening the physical and mental condition of the participant.”
4. Medical progress is based on research, which ultimately must rest in part on experimentation involving human subjects.
5. In medical research on human subjects, considerations related to the well being of the human subject should take precedence over the interests of science and society.
6. The primary purpose of medical research involving human subjects is to improve prophylactic, diagnostic and therapeutic procedures and the understanding of the aetiology and pathogenesis of the disease. Even the best-proven prophylactic, diagnostic and therapeutic methods must continuously be challenged through research for their effectiveness, efficiency, accessibility and quality.
7. In current medical practice and in medical research, most prophylactic, diagnostic and therapeutic procedures involve risks and burdens.
8. Medical research is subject to ethical standards that promote respect for all human beings and protect their health and rights. Some research populations are vulnerable and need special protection. The particular needs of the economically and medically disadvantaged must be recognised. Special attention is also required for those who cannot give or refuse consent for themselves, for those who may be subject to giving consent under duress, for those who will not benefit personally from the research and for those for whom the research is combined with care.
9. Research Investigators should be aware of the ethical, legal and regulatory requirements for research on human subjects in their own countries as well as applicable international requirements. No national ethical, legal or regulatory requirement should be allowed to reduce or eliminate any of the protections for human subjects set forth in this Declaration.

**B. Basic Principles for all Medical Research**

1. It is the duty of the physician in medical research to protect the life, health, privacy and dignity of the human subject.
2. Medical Research involving human subjects must conform to generally accepted scientific principles, be based on a thorough knowledge of the scientific literature, other relevant sources of information and on adequate laboratory and, where appropriate, animal experimentation.
3. Appropriate caution must be exercised in the conduct of research, which may affect the environment, and the welfare of animals used for research must be respected.
4. The design and performance of each experimental procedure involving human subjects should be clearly formulated in an experimental protocol. This protocol should be submitted for consideration, comment, guidance and, where appropriate, approval to a specially appointed ethical review committee, which must be independent of the investigator, the sponsor or any other kind of undue influence. This independent committee should be in conformity with the laws and regulations of the country in which the research experiment is performed. The committee has the right to monitor ongoing trials. The researcher has the obligation to provide monitoring information to the committee, especially any serious adverse events.
5. The researcher should also submit to the committee, for review, information regarding funding, sponsors, institutional affiliations, other potential conflicts of interest and incentives for subjects.
6. The research protocol should always contain a statement of the ethical considerations involved and should indicate that there is compliance with the principles enunciated in this Declaration.
7. Medical research involving human subjects should be conducted only by scientifically qualified persons and under the supervision of a clinically competent medical person. The responsibility for the human subject must always rest with a medically qualified person and never rest on the subject of the research, even though the subject has given consent.
8. Every medical research project involving human subjects should be preceded by careful assessment of predictable risks and burdens in comparison with foreseeable benefits to the subject or to others. This does not preclude the participation of healthy volunteers in medical research. The design of all studies should be publicly available.
9. Physicians should abstain from engaging in research projects involving human subjects unless they are confident that the risks involved have been adequately assessed and can be satisfactorily managed. Physicians should cease any investigation if the risks are found to outweigh the potential benefit or if there is conclusive proof of positive and beneficial results.
10. Medical research involving human subjects should only be conducted if the importance of the objective outweighs the inherent risks and burdens to the subject. This is especially important when the human subjects are healthy volunteers.
11. Medical research is only justified if there is a reasonable likelihood that the populations in which the research is carried out stand to benefit from the results of the research.
12. The subjects must be volunteers and informed participants in the research project.
13. The right of research subjects to safeguard their integrity must always be respected. Every precaution should be taken to respect the privacy of the subject, the confidentiality of the participant’s information and to minimise the impact of the study on the subject’s physical and mental integrity and on the personality of the subject.
14. In any research on human beings, each potential subject must be adequately informed of the aims, methods, sources of funding, any possible conflicts of interest, institutional affiliations of the researcher, the anticipated benefits and potential risks of the study and the discomfort it may entail. The subject should be informed of the right to abstain from participation in the study or to withdraw consent to participate at any time without reprisal. After ensuring that the subject has understood the information, the physician should then obtain the subject freely given informed consent, preferably in writing. If the consent cannot be obtained in writing, the non-written consent must be formally documented and witnessed.
15. When obtaining informed consent for the research project the physician should be particularly cautious if the subject is in a dependent relationship with the physician or may consent under duress. In that case, the informed consent should be obtained by a well-informed physician who is not engaged in the investigation and who is completely independent of this relationship.
16. For a research subject who is legally incompetent, physically or mentally incapable of giving consent or is a legally incompetent minor, the investigator must obtain informed consent from the legally authorised representative in accordance with applicable law. These groups should not be included in research unless the research is necessary to promote the health of the population represented and this research cannot instead be performed on legally competent persons.
17. When a subject deemed legally incompetent, such as a minor child, is able to give assent to decisions about participation in research, the investigator must obtain that assent in addition to the consent of the legally authorised representative.
18. Research on individuals from whom it is not possible to obtain consent, including proxy or advance consent, should be done only if the physical/mental condition that prevents obtaining informed consent is a necessary characteristic of the research population. The specific reasons for involving research subjects with a condition that renders them unable to give informed consent should be stated in the experimental protocol for consideration and approval of the review committee. The protocol should state that consent to remain in the research should be obtained as soon as possible from the individual or a legally authorised surrogate.
19. Both authors and publishers have ethical obligations. In publication of the results of research, the investigators are obliged to preserve the accuracy of the results. Negative as well as positive results should be published or otherwise publicly available. Sources of funding, institutional affiliations and any possible conflicts of interest should be declared in the publication. Reports of experimentation not in accordance with the principles laid down in this Declaration should not be accepted for publication.

**C. Additional Principles for Medical Research Combined with Medical Care**

1. The physician may combine medical research with medical care, only to the extent that the research is justified by its potential prophylactic, diagnostic or therapeutic value. When medical research is combined with medical care, additional standards apply to protect the participants who are research subjects.
2. The benefits, risks, burdens and effectiveness of a new method should be tested against those of the best current prophylactic, diagnostic and therapeutic methods. This does not exclude the use of placebo or no treatment, in studies where no proven prophylactic, diagnostic or therapeutic method exists.
3. At the conclusion of the study, every participant entered into the study should be assured of access to the best-proven prophylactic, diagnostic and therapeutic methods identified by the study.
4. The physician should fully inform the participant which aspects of the care are related to the research. The refusal of a participant to participate in a study must never interfere with the participant-physician relationship.
5. In the treatment of a participant, where proven prophylactic, diagnostic and therapeutic methods do not exist or have been ineffective, the physician, with informed consent from the participant, must be free to use unproven or new prophylactic, diagnostic and therapeutic measures, if in the physician’s judgement it offers hope of saving life, re-establishing health or alleviating suffering. Where possible, these measures should be made the object of research, designed to evaluate their safety and efficacy. In all cases, new information should be recorded and, where appropriate, published. The other relevant guidelines of this Declaration should be followed.

Appendix 2: ICH guidelines on Good Clinical Practice

**Responsibilities of the Investigator**

**Investigator’s Qualifications and Agreements**

1. The investigator(s) should be qualified by education, training, and experience to assume responsibility for the proposed conduct of the trial, should meet all the qualifications specified by the applicable regulatory requirement(s), and should provide evidence of such qualifications through up-to-date curriculum vitae and/or other relevant documentation requested by the sponsor, the IRB/IEC, and/or the regulatory authority(ies).

2. The investigator should be thoroughly familiar with the appropriate use of the investigational product(s), as described in the protocol, in the current Investigator’s Brochure, in the product information and in other information sources provided by the sponsor.

3. The investigator should be aware of, and should comply with, GCP and the applicable regulatory requirements.

4. The investigator/institution should permit monitoring and auditing by the sponsor, and inspection by the appropriate regulatory authority(ies).

5. The investigator should maintain a list of appropriately qualified persons to whom the investigator has delegated significant trial-related duties.

**Adequate Resources**

1. The investigator should be able to demonstrate (e.g. based on retrospective data) a potential for recruiting the required number of suitable subjects with the agreed recruitment period.

2. The investigator should have sufficient time to properly conduct and complete the trial within the agreed trial period.

3. The investigator should have available an adequate number of qualified staff and adequate facilities for the foreseen duration of the trial to conduct the trial properly and safely.

4. The investigator should ensure that all persons assisting with the trial are adequately informed about the protocol, the investigational product(s), and their trial-related duties and functions.

**Medical Care of Trial Subjects**

1. A qualified physician (or dentist, when appropriate), who is an investigator or a sub-investigator for the trial, should be responsible for all trial-related medical (or dental) decisions.

2. During and following a subject’s participation in a trial, the investigator/institution should ensure that adequate medical care is provided to a subject for any adverse events, including clinically significant laboratory values, related to the trial. The investigator/institution should inform a subject when medical care is needed for intercurrent illness(es) of which the investigator becomes aware.

3. It is recommended that the investigator inform the subject’s primary physician about the subject’s participation in the trial if the subject has a primary physician and if the subject agrees to the primary physician being informed.

4. Although a subject is not obliged to give his/her reason(s) for withdrawing prematurely from a trial, the investigator should make a reasonable effort to ascertain the reason(s), while fully respecting the subject’s rights.

**Communication with IRB/IEC**

1. Before initiating a trial, the investigator/institution should have written and dated approval/favourable opinion from the IRB/IEC for the trial protocol, written informed consent form, consent form updates, subject recruitment procedures (e.g. advertisements) and any other written information to be provided to subjects.

2. As part of the investigator’s/institution’s written application to the IRB/IEC, the investigator/institution should provide the IRB/IEC with a current copy of the Investigator’s Brochure. If the Investigator’s Brochure is updated during the trial, the investigator/institution should supply a copy of the updated Investigator’s Brochure to the IRB/IEC.

3. During the trial, the investigator/institution should provide to the IRB/IEC all documents subject to review.

**Compliance with Protocol**

1. The investigator/institution should conduct the trial in compliance with the protocol agreed to by the sponsor and, if required, by the regulatory authority(ies) and which was given approval/favourable opinion by the IRB/IEC. The investigator/institution and the sponsor should sign the protocol, or an alternative contract, to confirm agreement.

2. The investigator should not implement any deviation from, or changes of the protocol without agreement by the sponsor and prior review and documented approval/ favourable opinion from the IRB/IEC of an amendment, except where necessary to eliminate an immediate hazard(s) to trial subjects, or when the change(s) involves only logistical or administrative aspects of the trial (e.g. change in monitor(s), change of telephone number(s).

3. The investigator, or person designated by the investigator, should document and explain any deviation from the approved protocol.

4. The investigator may implement a deviation from, or a change of, the protocol to eliminate an immediate hazard(s) to trial subjects without prior IRB/IEC approval/ favourable opinion. As soon as possible, the implemented deviation or change, the reasons for it, and, if appropriate, the proposed protocol amendment(s) should be submitted:

1. to the IRB/IEC for review and approval/favourable opinion,
2. to the sponsor for agreement and, if required,
3. to the regulatory authority(ies).

**Investigational Product(s)**

1. Responsibility for investigational product(s) accountability at the trial site(s) rests with the investigator/institution.

2. Where allowed/required, the investigator/institution may/should assign some or all the investigator’s/institution’s duties for investigational product(s) accountability at the trial site(s) to an appropriate pharmacist or another appropriate individual who is under the supervision of the investigator/institution.

3. The investigator/institution and/or a pharmacist or other appropriate individual, who is designated by the investigator/institution should maintain records of the product’s delivery to the trial site, the inventory at the site, the use by each subject, and the return to the sponsor or alternative disposition of unused product(s). These records should include dates, quantities, batch/serial numbers, expiration dates (if applicable), and the unique code numbers assigned to the investigational product(s) and trial subjects. Investigators should maintain records that document adequately that the subjects were provided the doses specified by the protocol and reconcile all investigational product(s) received from the sponsor.

4. The investigational product(s) should be stored as specified by the sponsor and in accordance with applicable regulatory requirement(s).

5. The investigator should ensure that the investigational product(s) are used only in accordance with the approved protocol.

6. The investigator, or a person designated by the investigator/institution, should explain the correct use of the investigational product(s) to each subject and should check, at intervals appropriate for the trial, that each subject is following the instructions properly.

**Randomisation Procedures and Unblinding**

1. The investigator should follow the trial’s randomisation procedures, if any, and should ensure that the code is broken only in accordance with the protocol. If the trial is blinded, the investigator should promptly document and explain to the sponsor any premature unblinding (e.g. accidental unblinding, unblinding due to a serious adverse event) of the investigational product(s).

**Informed Consent of Trial Subjects**

1. In obtaining and documenting informed consent, the investigator should comply with the applicable regulatory requirement(s), and should adhere to GCP and to the ethical principles that have their origin in the Declaration of Helsinki. Before the beginning of the trial, the investigator should have the IRB/IEC’s written approval/favourable opinion of the written informed consent form and any other written information to be provided to subjects.

2. The written informed consent form and any other written information to be provided to subjects should be revised whenever important new information becomes available that may be relevant to the subject’s consent. Any revised written informed consent form, and written information should receive the IRB/IEC’s approval/favourable opinion in advance of use. The subject or the subject’s legally acceptable representative should be informed in a timely manner if new information becomes available that may be relevant to the subject’s willingness to continue participation in the trial. The communication of this information should be documented.

3. Neither the investigator, nor the trial staff, should coerce or unduly influence a subject to participate or to continue to participate in a trial.

4. None of the oral and written information concerning the trial, including the written informed consent form, should contain any language that causes the subject or the subject’s legally acceptable representative to waive or to appear to waive any legal rights, or that releases or appears to release the investigator, the institution, the sponsor, or their agents from liability for negligence.

5. The investigator, or a person designated by the investigator, should fully inform the subject or, if the subject is unable to provide informed consent, the subject’s legally acceptable representative, of all pertinent aspects of the trial including the written information given approval/favourable opinion by the IRB/IEC.

6. The language used in the oral and written information about the trial, including the written informed consent form, should be as non-technical as practical and should be understandable to the subject or the subject’s legally acceptable representative and the impartial witness, where applicable.

7. Before informed consent may be obtained, the investigator, or a person designated by the investigator, should provide the subject or the subject’s legally acceptable representative ample time and opportunity to enquire about details of the trial and to decide whether or not to participate in the trial. All questions about the trial should be answered to the satisfaction of the subject or the subject’s legally acceptable representative.

8. Prior to a subject’s participation in the trial, the written informed consent form should be signed and personally dated by the subject or the subject’s legally acceptable representative, and by the person who conducted the informed consent discussion.

9. If a subject is unable to read or if a legally acceptable representative is unable to read, an impartial witness should be present during the entire informed consent discussion. After the written informed consent form and any other written information to be provided to subjects, is read and explained to the subject or the subject’s legally acceptable representative, and after the subject or the subject’s legally acceptable representative has orally consented to the subject’s participation in the trial and, if capable of doing so, has signed and personally dated the informed consent form, the witness should sign and personally date the consent form. By signing the consent form, the witness attests that the information in the consent form and any other written information was accurately explained to, and apparently understood by, the subject or the subject’s legally acceptable representative, and that informed consent was freely given by the subject or the subject’s legally acceptable representative.

10. Both the informed consent discussion and the written informed consent form and any other written information to be provided to subjects should include explanation of the following:

1. That the trial involves research.
2. The purpose of the trial.
3. The trial treatment(s) and the probability for random assignment to each treatment.
4. The trial procedures to be followed, including all invasive procedures.
5. The subject’s responsibilities.
6. Those aspects of the trial that are experimental.
7. The reasonably foreseeable risks or inconveniences to the subject and, when applicable, to an embryo, foetus, or nursing infant.
8. The reasonably expected benefits. When there is no intended clinical benefit to the subject, the subject should be made aware of this.
9. The alternative procedure(s) or course(s) of treatment that may be available to the subject, and their important potential benefits and risks.
10. The compensation and/or treatment available to the subject in the event of trial-related injury.
11. The anticipated prorated payment, if any, to the subject for participating in the trial.
12. The anticipated expenses, if any, to the subject for participating in the trial.
13. That the subject’s participation in the trial is voluntary and that the subject may refuse to participate or withdraw from the trial, at any time, without penalty or loss of benefits to which the subject is otherwise entitled.
14. That the monitor(s), the auditor(s), the IRB/IEC, and the regulatory authority(ies) will be granted direct access to the subject’s original medical records for verification of clinical trial procedures and/or data, without violating the confidentiality of the subject, to the extent permitted by the applicable laws and regulations and that, by signing a written informed consent form, the subject or the subject’s legally acceptable representative is authorising such access.
15. That records identifying the subject will be kept confidential and, to the extent permitted by the applicable laws and/or regulations, will not be made publicly available. If the results of the trial are published, the subject’s identity will remain confidential.
16. That the subject or the subject’s legally acceptable representative will be informed in a timely manner if information becomes available that may be relevant to the subject’s willingness to continue participation in the trial.
17. The person(s) to contact for further information regarding the trial and the rights of trial subjects, and whom to contact in the event of trial-related injury.
18. The foreseeable circumstances and/or reasons under which the subject’ s participation in the trial may be terminated.
19. The expected duration of the subject’s participation in the trial.
20. The approximate number of subjects involved in the trial.

11. Prior to participation in the trial, the subject or the subject’s legally acceptable representative should receive a copy of the signed and dated written informed consent form and any other written information provided to the subjects. During a subject’s participation in the trial, the subject or the subject’s legally acceptable representative should receive a copy of the signed and dated consent form updates and a copy of any amendments to the written information provided to subjects.

12. When a clinical trial (therapeutic or non-therapeutic) includes subjects who can only be enrolled in the trial with the consent of the subject’s legally acceptable representative (e.g. minors, or participants with severe dementia), the subject should be informed about the trial to the extent compatible with the subject’s understanding and, if capable, the subject should sign and personally date the written informed consent.

13. Except as described in 14, a non-therapeutic trial (i.e. a trial in which there is no anticipated direct clinical benefit to the subject), should be conducted in subjects who personally give consent and who sign and date the written informed consent form.

14. Non-therapeutic trials may be conducted in subjects with consent of a legally acceptable representative provided the following conditions are fulfilled:

1. The objectives of the trial cannot be met by means of a trial in subjects who can give informed consent personally.
2. The foreseeable risks to the subjects are low.
3. The negative impact on the subject’s well being is minimised and low.
4. The trial is not prohibited by law.
5. The approval/favourable opinion of the IRB/IEC is expressly sought on the inclusion of such subjects, and the written approval/favourable opinion covers this aspect.

Such trials, unless an exception is justified, should be conducted in participants having a disease or condition for which the investigational product is intended. Subjects in these trials should be particularly closely monitored and should be withdrawn if they appear to be unduly distressed.

15. In emergency situations, when prior consent of the subject is not possible, the consent of the subject’s legally acceptable representative, if present, should be requested. When prior consent of the subject is not possible, the subject’s legally acceptable representative is not available, enrolment of the subject should require measures described in the protocol and/or elsewhere, with documented approval/favourable opinion by the IRB/IEC, to protect the rights, safety and well being of the subject and to ensure compliance with applicable regulatory requirements. The subject or the subject’s legally acceptable representative should be informed about the trial as soon as possible and consent to continue and other consent as appropriate (see 10) should be requested.

**Records and Reports**

1. The investigator should ensure the accuracy, completeness, legibility, and timeliness of the data reported to the sponsor in the CRFs and in all required reports.

2. Data reported on the CRF that are derived from source documents should be consistent with the source documents or the discrepancies should be explained.

3. Any change or correction to a CRF should be dated, initialled, and explained (if necessary) and should not obscure the original entry (i.e. an audit trail should be maintained); this applies to both written and electronic changes or corrections. Sponsors should provide guidance to investigators and/or the investigator’s designated representatives on making such corrections.

Sponsors should have written procedures to assure that changes or corrections in CRFs made by sponsor’s designated representatives are documented, are necessary and are endorsed by the investigator. The investigator should retain records of the changes and corrections.

4. The investigator/institution should maintain the trial documents as specified in Essential Documents for the Conduct of a Clinical Trial and as required by the applicable regulatory requirement(s). The investigator/institution should take measures to prevent accidental or premature destruction of these documents.

5. Essential documents should be retained until at least 2 years after the last approval of a marketing application in an ICH region and until there are no pending or contemplated marketing applications in an ICH region or at least 2 years have elapsed since the formal discontinuation of clinical development of the investigational product. These documents should be retained for a longer period however if required by the applicable regulatory requirements or by an agreement with the sponsor. It is the responsibility of the sponsor to inform the investigator/institution as to when these documents no longer need to be retained.

6. The financial aspects of the trial should be documented in an agreement between the sponsor and the investigator/institution.

7. Upon request of the monitor, auditor, IRB/IEC or regulatory authority, the investigator/institution should make available for direct access all requested trial-related records.

**Progress Reports**

1. The investigator should submit written summaries of the trial status to IRB/IEC annually, or more frequently, if requested by the IRB/IEC.

2. The investigator should promptly provide written reports to the sponsor, the IRB/IEC and, where applicable, the institution on any changes significantly affecting the conduct of the trial, and/or increasing the risk to subjects.

**Safety Reporting**

1. All serious adverse events (SAEs) should be reported immediately to the sponsor except for those SAEs that the protocol or other document (e.g. Investigator’s Brochure) identifies as not needing immediate report. The immediate reports should be followed promptly by detailed, written reports. The immediate and follow-up reports should identify subjects by unique code numbers assigned to the trial subjects rather than by the subjects’ names, personal identification numbers and/or addresses. The investigator should also comply with the applicable regulatory requirement(s) related to the reporting of unexpected serious adverse drug reactions to the regulatory authority(ies) and the IRB/IEC.

2. Adverse events and/or laboratory abnormalities identified in the protocol as critical to safety evaluations should be reported to the sponsor according to the reporting requirements and within the time periods specified by the sponsor in the protocol.

3. For reported deaths, the investigator should supply the sponsor and the IRB/IEC with any additional requested information (e.g. autopsy reports and terminal medical reports).

**Premature Termination or Suspension of a Trial**

1. If the trial is prematurely terminated or suspended for any reason, the investigator/ institution should promptly inform the trial subjects, should assure appropriate therapy and follow-up for the subjects, and, where required by the applicable regulatory requirement(s), should inform the regulatory authority(ies). In addition to:

a) If the investigator terminates or suspends a trial without prior agreement of the sponsor, the investigator should inform the institution where applicable, and the investigator/institution should promptly inform the sponsor and the IRB/IEC, and should provide the sponsor and the IRB/IEC a detailed written explanation of the termination or suspension.

b) If the sponsor terminates or suspends a trial, the investigator should promptly inform the institution where applicable and the investigator/institution should promptly inform the IRB/IEC and provide the IRB/IEC a detailed written explanation of the termination or suspension.

c) If the IRB/IEC terminates or suspends its approval/favourable opinion of a trial, the investigator should inform the institution where applicable and the investigator/institution should promptly notify the sponsor and provide the sponsor with a detailed written explanation of the termination or suspension.

**Final Report(s) by Investigator**

1. Upon completion of the trial, the investigator, where applicable, should inform the institution; the investigator/institution should provide the IRB/IEC with a summary of the trial’s outcome, and the regulatory authority(ies) with any reports required.
