# Supplementary material for: Randomised, Controlled, Assessor Blind Trial Comparing 4% Dimeticone Lotion with 0.5% Malathion Liquid for Head Louse Infestation
Source: PLoS One. 2007 Nov 7;2(11):e1127. doi: 10.1371/journal.pone.0001127 (PMC2043492; doi:10.1371/journal.pone.0001127)
Supplement: Table S1 — Treatment outcome for incorrectly randomised participants (0.03 MB DOC) [file pone.0001127.s003.doc]

Table S1 Treatment outcome for incorrectly randomised participants

| **Participant number** | **Treatment outcome** | **Comments** |
| --- | --- | --- |
| 017 | Treatment failure | Nymph after 2^nd^ treatment |
| 019 | Drop out | Family problems |
| 021 | Cure | - |
| 023 | Cure | - |
| 060 | Cure | - |
| 061 | Treatment failure | - |
| 062 | Cure | - |
