# Supplementary material for: Randomised, Controlled, Assessor Blind Trial Comparing 4% Dimeticone Lotion with 0.5% Malathion Liquid for Head Louse Infestation
Source: PLoS One. 2007 Nov 7;2(11):e1127. doi: 10.1371/journal.pone.0001127 (PMC2043492; doi:10.1371/journal.pone.0001127)
Supplement: Table S2 — Analysis of cases of treatment failure following 4% dimeticone lotion treatment (0.04 MB DOC) [file pone.0001127.s004.doc]

Table S2 Analysis of cases of treatment failure following 4% dimeticone lotion treatment

| **Study number** | **Outcome** | **Lice found *** | | **Family members in the study** | **Possible explanation** |
| --- | --- | --- | --- | --- | --- |
|  |  | Day 9 | Day 14 |  |  |
| 004 | Treatment failure | 1x ♀ | 1x #2 | 003 TF **  005 TF | Reinfestation within family |
| 011 | Ovicidal failure | 1x #1 |  | 010 TF  016 TF | Reinfestation within family |
| 017 | Ovicidal failure |  | 1x #2 | 018 R  021 C | Delayed egg hatch |
| 042 | Treatment failure | 1x ♂  2x ♂  1x #3 | 3x ♂  2x ♀ | 043 R  044 C | Failure of treatment |
| 051 | Ovicidal failure |  | 2x #1 | 068 R  069 R  070 TF | Delayed egg hatch |
| 052 | Ovicidal failure | 1x #1 |  | 053 C | Delayed egg hatch |
| 061 | Treatment failure | 1x #2 | 3x ♂  1x #3  2x #1 | 060 C  062 C | Failure of treatment |
| 067 | Ovicidal failure | 1x #1 |  | 077 C  079 R  080 TF | Delayed egg hatch |
| 080 | Ovicidal failure | 1x #1 |  | 067 TF  077 C  079 R | Delayed egg hatch |

* ♀ = adult female, ♂= adult male, #3 = third stage nymph, #2 = second stage nymph, #1 = first stage nymph

** TF = treatment failure, R = reinfestation, C = cure
