# Supplementary material for: Randomised, Controlled, Assessor Blind Trial Comparing 4% Dimeticone Lotion with 0.5% Malathion Liquid for Head Louse Infestation
Source: PLoS One. 2007 Nov 7;2(11):e1127. doi: 10.1371/journal.pone.0001127 (PMC2043492; doi:10.1371/journal.pone.0001127)
Supplement: Table S3 — Adverse events as recorded on the case report form (0.05 MB DOC) [file pone.0001127.s005.doc]

Table S3 Adverse events as recorded on the case report form

| Participant | Adverse Event | Timing* | Severity | Action Taken | Relationship to  Study Product | Outcome+ |
| --- | --- | --- | --- | --- | --- | --- |
|  |  |  |  |  |  |  |
| Malathion | |  |  |  |  |  |
| 008 | Toothache | 1+ | Mild | Medication | Unrelated | Resolved |
| 028 | Head sore/stinging | 1 | Mild | None | Possible | Resolved |
| 035 | Sore throat and cold | 1+ | Mild | None | Unrelated | Resolved |
| 045 no. 1 | Burning sensation to scalp | 1 | Mild | None | Probable | Resolved |
| 045 no. 2 | Burning sensation to scalp | 2 | Mild | None | Probable | Resolved |
| 047 | Fell at school, grazed hand | 1+ | Mild | None | Unrelated | Resolved |
| 054 | Stuffy nose/throat | 1+ | Mild | Medication | Unrelated | Resolved |
| 056 | Sore throat and cold | 1+ | Mild | Medication | Unrelated | Resolved |
| 057 | Sore throat | 1+ | Mild | None | Unrelated | Resolved |
| 076 | Fell at school, banged knee | 1+ | Mild | None | Unrelated | Resolved |
|  |  |  |  |  |  |  |
| Dimeticone | |  |  |  |  |  |
| 001 | Rash back of neck | 2+ | Mild | None | Unknown | Resolved |
| 019 | Otitis media | 1+ | Moderate | Medication | Unrelated | Ongoing |
| 033 | Cold | 2+ | Mild | None | Remote | Ongoing |
| 052 | Cat scratch | 2+ | Mild | None | Unrelated | Resolved |
| 069 | Otitis media | 2 | Mild | Medication | Remote | Ongoing |
|  |  |  |  |  |  |  |

* **1** = onset on day of first application;

**2** = onset on day of second application;

**1+** = onset after first and before second application;

**2+** = onset after second application

+ “Resolved” indicates that it had resolved by the time the event was first recorded

“Ongoing” indicates that it had not resolved by the time the event was first recorded
